# Relevance of oncobiome in breast cancer evolution in an Argentine cohort

**DOI:** 10.1128/msphere.00597-24

**Published:** 2025-02-10

**Authors:** Leonardo Néstor Rubén Dandeu, Joel Lachovsky, Sofía Sidlik, Pablo Marenco, Daniela Orschanski, Pablo Aguilera, Martín Vázquez, María del Pilar Carballo, Elmer Fernández, Alberto Penas-Steinhardt, Norma Alejandra Chasseing, Vivian Labovsky

**Affiliations:** 1 Laboratorio de Inmunohematología, Instituto de Biología y Medicina Experimental (IBYME), Fundación IBYME, Consejo Nacional de Investigaciones Científicas y Técnicas (CONICET), Ciudad Autónoma de Buenos Aires, Buenos Aires, Argentina; 2 Instituto de Oncología Ángel H Roffo, Ciudad Autónoma de Buenos Aires, Buenos Aires, Argentina; 3 Fundación para el Progreso de la Medicina 637357 , Córdoba, Argentina; 4 Departamento de Ciencias Aplicadas y Tecnología, Universidad Nacional de Moreno 441449 , Moreno, Argentina; 5 Instituto Héritas, Rosario, Argentina; 6 Departamento de Ciencias Básicas, Laboratorio de Genómica Computacional (GEC-UNLu), Universidad Nacional de Luján, Luján, Argentina; Kansas State University, Manhattan, Kansas, USA

**Keywords:** tumor microenvironment, oncobiome, breast cancer, progression disease

## Abstract

Breast cancer is the leading cause of cancer deaths in women worldwide, with about 20,000 cases annually in Argentina. While age, diet, and genetics are known risk factors, most breast cancer cases have unknown causes, necessitating the discovery of new risk factors. The aim of this study was the analysis of the prognostic relevance of the oncobiome in Argentinean breast cancer patients. Sequencing of the V4 region 16S rRNA gene was performed on 34 primary breast tumor samples, using bioinformatic and statistical analyses to identify bacteria and hypothetical pathways. Each sample presented a unique microbial profile, with *Proteobacteria* being the most abundant phylum. Tumors >2 cm showed greater alpha diversity with increased nucleotide biosynthesis. Moreover, progesterone-receptor tumors showed differences in beta diversity, being progesterone receptor-positive tumors that had the highest expression of *Acinetobacter* and *Moraxella*. In disease progression, the phylum *Chloroflexi* was prevalent in tumors of surviving patients. *Acinetobacter* and *Cloacibacterium* genera were significantly higher in patients without events and those without metastasis. We found that nucleotide and cell-structure biosynthesis, and lipid metabolism pathways were enriched in tumors with poor progression, whereas amino-acid degradation was increased in tumors of surviving patients. This finding is an indication that tumor cells are taking advantage of this effect of the microbiome during tumor progression. We conclude that oncobiome is dysbiotic in these patients, with distinct patterns in those with poor progression. Suggesting a link between the oncobiome and cancer progression, paving the way for new therapies to improve patient quality of life and survival.

**IMPORTANCE:**

This is the first study to investigate the relevance of the oncobiome in the evolution of breast cancer in a cohort of Argentine patients. It also highlights the need for further research in this area to improve our understanding of the role of the microbiome in this disease and potentially identify new therapeutic targets or prognostic indicators. Understanding the complex interaction between the microbiome, the tumor microenvironment, and the pathogenesis of breast cancer holds the promise of more personalized and effective treatment approaches in the future.

## INTRODUCTION

Cancer is one leading cause of death before the age of 70 in 112 countries around the world. In Latin America and the Caribbean cancer accounts for more than 210,000 new cases and about 60,000 deaths per year. Particularly, breast cancer (BC) is the primary cause of death for women worldwide according to the number of new cases and deaths estimated by the American Cancer Society (1). In South America, Argentina has the second highest mortality rate with 6,380 deaths per year (2). Breast cancer is classified into four main subtypes (3) based on hormone receptors estrogen receptor (ER), progesterone receptor (PR), and human epidermal growth factor receptor 2 (HER2/neu) status, as follows: (i) luminal A: ER+ and/or PR+, HER2/neu-negative (slow-growing, good response to hormonal therapy, and better prognosis); (ii) luminal B: ER+ and/or PR+, HER2/neu-positive or negative (faster-growing and more aggressive, often needs both hormonal therapy and chemotherapy); (iii) HER2/neu-enriched: ER- and PR-negative, HER2/neu-positive (rapid growth, but responds well to HER2/neu-targeted treatments); and (iv) triple-negative (TNBC): ER-, PR-, HER2/neu-negative (most aggressive, requiring chemotherapy as it does not respond to hormonal or HER2 therapies).

From a clinical perspective, hormone receptors, HER2/neu, and the Ki-67 antigen are considered prognostic biomarkers. Measuring these biomarkers helps determine the most appropriate hormonal and anti-HER2/neu antibody therapy, if necessary. In addition to this, surgery, radiotherapy, chemotherapy, and immunotherapy are current BC treatment strategies. However, patient responses to treatment are not identical, indicating the heterogeneity of individuals, BC characteristics, and the tumor microenvironment (3). There is evidence that tumor cells and their microenvironments regulate disease progression. The breast tumor microenvironment consists of various cellular components, including endothelial cells, pericytes, tumor-associated macrophages, immune cells, adipocytes, fibroblasts, cancer-associated fibroblasts, mesenchymal stromal/stem cells, hematopoietic stem cells, and myeloid-derived suppressor cells. Additionally, it contains soluble factors such as cytokines, chemokines, growth factors, metalloproteinases, tissue inhibitors of metalloproteinases, and extracellular matrix components (4–6). Moreover, the microbiome is increasingly recognized as a key feature of both the tumor tissue and the tumor microenvironment (7, 8). It has been demonstrated that the microbiome interacts with the host, affecting its physiology and predisposing it to the development of various diseases (9). Despite the high incidence of BC, many patients do not exhibit known risk factors, making it extremely important to identify new tumor-associated risk factors. The microbiome is known to influence tumor progression through various mechanisms, including DNA damage, oncogenic pathway activation, modulation of anti-tumor immunity, and alteration of drug metabolism (e.g., chemotherapeutics) (10).

Dysbiosis is defined as the abnormal adaptation of the microbiome and is characterized by an altered microbial and functional composition. The microbiome undergoing oncobiotic transformations is referred to as the oncobiome (11).

Given the necessity to gather evidence on the relevance of oncobiome in BC progression and the lack of research in this area, we conducted a study on a cohort of patients from Argentina.

## MATERIALS AND METHODS

### Patient samples

To investigate the prognostic relevance of the microbiome in the evolution of BC, a retrospective study was carried out including biopsies of frozen breast tumor tissue. This study included 34 consecutive cases with a confirmed histological diagnosis made by pathologists. Tumor samples and clinical data were obtained from women (range of age 18–90) undergoing breast surgical resection at the Instituto de Oncología Ángel Roffo (Buenos Aires, Argentina). After excision, fresh breast tumor tissues were immediately placed in a sterile vial on ice and frozen at −70°C. Inclusion criteria included women with invasive ductal breast carcinoma and clinicopathological stages I, II, III (early, *N* = 32), and IV (advanced, *N* = 2), according to the International Union Against Cancer TNM classification system. The exclusion criteria comprised neoadjuvant therapies, previous development of another primary tumor, and/or underlying immune diseases. The analysis of clinicopathological features of BC and those associated with patient lifestyles was conducted on the total sample (*N* = 34). Analysis of disease progression features (occurrence of any event, including relapse, metastasis, and death), was only performed on samples from patients with a minimum follow-up of 5 years post-surgery (*N* = 27). The samples were subdivided into groups: (i) <50 and ≥50 years old; (ii) tumor size ≤2 and >2 cm; (iii) stage (early/advanced); (iv) histological grade was evaluated based on the Scarff-Bloom-Richardson grading system and categorized as well differentiated/intermediate (G1/G2) and poor (G3); (v) HER2/neu and hormonal receptors (ER/PR) expression according to Wernicke et al. (12); (vi) ki-67 cell marker expression was considered <15 negative and ≥15 positive; (vii) molecular subtype was categorized as well as luminal A, luminal B, HER2-positive, and triple-negative (TNBC); (viii) presence of regional metastatic lymph nodes was recorded as negative (negative nodes in axillary dissection or negative sentinel lymph node when this technique was performed) or positive (including micrometastasis); (ix) smoke consumption, (x) alcohol consumption; (xi) pregnancy; (xii) lactation and (xiii) menstrual status; (xiv) event; (xv) metastatic event; and (xvi) death event. Patient features are shown in Tables 1 to 3.

**TABLE 1:** Clinicopathological features of 34 patients with invasive ductal breast cancer^*a*^

Clinicopathological feature	Patients (*N*)	Patients (%)
Age (years)		
<50	8	23.53
≥50	26	76.47
Unknown	0	0
Tumor size (cm)		
≤2	11	32.35
>2	23	67.65
Unknown	0	0
Stage		
Early	32	94.12
Advanced	2	5.88
Unknown	0	0
Histological grade		
G1	7	20.59
G2	14	41.18
G3	13	38.24
Unknown	0	0
Her2/neu positive		
No	26	76.47
Yes	8	23.53
Unknown	0	0
ER positive		
No	5	14.71
Yes	29	85.29
Unknown	0	0
PR positive		
No	10	29.41
Yes	24	70.59
Unknown	0	0
Ki-67 status		
Negative (<15)	3	8.82
Positive (≥15)	31	91.18
Unknown	0	0
Molecular subtype		
Luminal A	22	64.71
Luminal B	7	20.59
HER2-positive	1	2.94
TNBC	4	11.76
Unknown	0	0
Regional lymph node positive		
No	15	44.12
Yes	19	55.88
Unknown	0	0

^*a*^ ER, estrogen receptor; PR, progesterone receptor; Her2/neu, human epidermal growth factor receptor 2.

**TABLE 2:** Lifestyle-related features of 34 patients with invasive ductal breast cancer

Lifestyle-related feature	Patients (*N*)	Patients (%)
Smoke consumption		
No	26	76.47
Yes	8	23.53
Unknown	0	0
Alcohol consumption		
No	30	88.24
Yes	4	11.76
Unknown	0	0
Pregnancy status		
No	4	11.76
Yes	27	79.41
Unknown	3	8.82
Lactation status		
No	10	29.41
Yes	19	55.88
Unknown	5	14.71
Menstrual status		
Premenopausal	7	20.59
Postmenopausal	26	76.47
Unknown	1	2.94

**TABLE 3:** Evolution features of 27 patients with invasive ductal breast cancer

Evolution event	Patients (*N*)	Patients (%)
Event		
No	20	74.07
Yes	7	25.92
Unknown	0	0
Metastatic event		
No	21	77.78
Yes	6	22.22
Unknown	0	0
Death event		
No	23	85.18
Yes	4	14.81
Unknown	0	0

### Genomic DNA extraction from tumor breast tissue

Samples were processed and DNA was extracted using the QIAamp-DNA-mini-kit (Qiagen, 51306), following the manufacturer’s instructions. DNA quality (A260/280, A260/230) and concentration were determined by spectrophotometry (Thermo Fisher Scientific, Waltham, MA, USA, NanoDrop 2000c). The Genomic DNA samples were stored at −20°C.

### 16S rRNA gene sequence analysis

Genomic DNA isolated from the samples was subjected to amplification using MiSeq adapter sequences. Following the initial amplification, the PCR product was purified and promptly stored at −20°C. To minimize contamination from environmental bacterial DNA, all working tools (pipettes, plasticware, and non-enzymatic kit components) were UV-irradiated before use, and all PCR reactions were conducted in a laminar flow hood. In order to evaluate the background from the experimental samples, we carry out a sample negative control to determine what sequences are present. These negative controls were performed by replacing the DNA template with nuclease-free water. We checked the first amplification of the samples and the control without DNA with agarose gel. We have observed that the negative control has no amplification of the band while the samples have. We also proceeded to sequence both the negative controls and the samples. After sequencing, the negative control samples showed no reads, ensuring that no contaminants were introduced during the process. Additionally, some breast samples had read counts under 1,000, which were excluded from the study; only samples with read counts above were used in our analyses.

The microbiome was identified by constructing a library using the hyper-variable V4-V4 regions of the 16S rRNA gene following Illumina MiSeq methodology. Quality control of raw sequence data was conducted using FastQC, and Trimmomatic v0.32 was employed for trimming low-quality reads and demultiplexing. The resulting sequencing files were processed with QIIME2 to identify the Amplicon Sequence Variant (ASVs) and assign taxonomy to each sequence from each sample.

### Analysis of predicted metabolic functions

The PICRUSt software (Phylogenetic Investigation of Communities by Reconstruction of Unobserved States) was used for identifying hypothetical metabolic functions based on and KEGG database (Kyoto Encyclopedia of Genes and Genome). The MetaCyc database was used to obtain information about bacterial metabolic pathways.

### Statistical analysis

The relative abundances of the ASVs were examined using algorithms in R, utilizing packages such as phyloseq and microbiome utilities. Statistical significance, with a threshold set at a *P*-value < 0.05, was determined for variations in relative abundances across diverse taxonomic levels among samples representing distinct metadata groups. Wilcoxon and Kruskal tests were used for this analysis. Additionally, assessments of alpha diversity were undertaken, utilizing the indices richness (Chao 1), evenness, and Shannon. For beta diversity, the Bray-Curtis index was employed. Subsequently, these diversity metrics were compared using Wilcoxon and Kruskal tests for alpha diversity, and the Adonis metric for beta diversity.

## RESULTS

### Breast cancer patient features

This study included frozen breast tumor tissue from 34 patients. Clinicopathological and lifestyle (exposome) patient features are shown in Tables 1 and 2, respectively. The majority of the patients had no history of smoking or alcohol consumption, while more than half of the cohort had a history of pregnancy and/or lactation. The study on the relevance of the microbiome in the evolution of breast cancer included frozen breast tumor tissue from 27 patients with a minimum 5-year follow-up (Table 3).

### Taxonomic profile of patient breast tumor tissue

**Fig 1:**
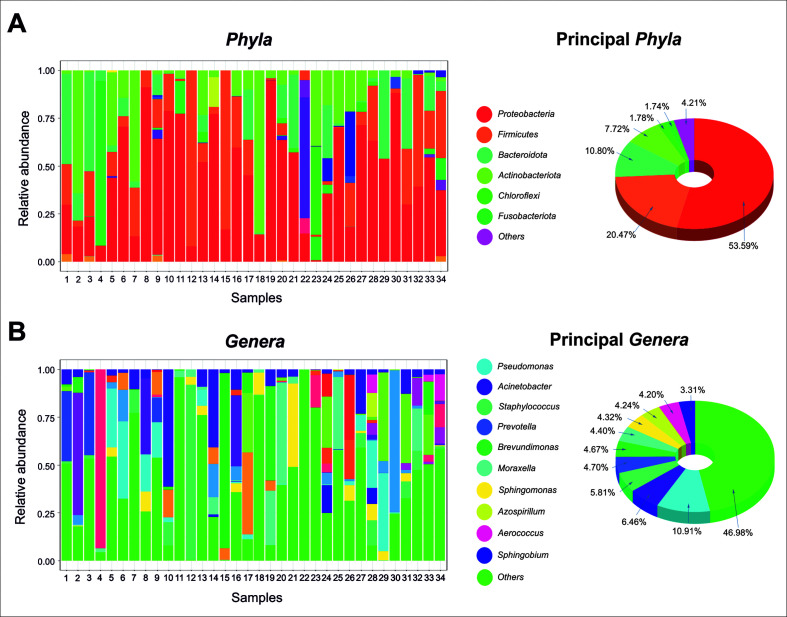
Each sample presented a distinctive microbial profile at phylum and genus levels (*N* = 34). (**A**) Relative abundances at the phylum level of all samples showed that *Proteobacteria* (53.59%), *Firmicutes* (20.47%), *Bacteroidota* (10.80%), and *Actinobacteriota* (7.41%) were the most abundant, representing more than 90% of the total. (**B**) Relative abundances at the genus level of all samples, showed the following 10 most abundant bacterial genera *Pseudomonas* (10.91%), *Acinetobacter* (6.46%), *Staphylococcus* (5. 81%), *Prevotella* (4.70%), *Brevundimonas* (4.67%), *Moraxella* (4.41%), *Sphingomonas* (4.32%), *Azospirillum* (4.24%), *Aerococcus* (4.20%), and *Sphingobium* (3.31%).

A total of 526 ASV and 409,710 reads were obtained. All samples had a minimum frequency of 1,000 reads. All of them were taxonomically annotated at least at the kingdom level. In total, 34 samples were analyzed. Each sample presented a distinctive microbial profile. The bacteria identified in the patient’s breast tumor tissue were presented from phylum to genus level. A total of 21 phyla, 33 classes, 73 orders, 128 families, and 207 genera were found. The most predominant phyla were *Proteobacteria* (53.59%), followed by *Firmicutes* (20.47%), *Bacteroidota* (10.80%), and *Actinobacteriota* (7.41%). The first four phyla collectively constituted more than 90% of the total. Completing the ranking of the top 10 most abundant phyla, we found *Chloroflexi* (1.78%), *Fusobacteriota* (1.74%), *Campylobacteriota* (1.21%), *Patescibacteria* (1.06%), *Verrucomicrobiota* (0.71%), and *Myxococcota* (0.45%) (Fig. 1A). At the genus level, the top 10 most abundant genera were *Pseudomonas* (10.91%), *Acinetobacter* (6.46%), *Staphylococcus* (5.81%), *Prevotella* (4.70%), *Brevundimonas* (4.67%), *Moraxella* (4.41%), *Sphingomonas* (4.32%), *Azospirillum* (4.24%), *Aerococcus* (4.20%), *Sphingobium* (3.31%), following by *Cloacibacterium* (2.77%), *Lactobacillus* (2.71%), *Escherichia-Shigella* (2.14%), *Bacillus* (2.10%), *Massilia* (2.09%), and others like *Fusobacterium* (1.79%), *Methylobacterium-Methylorubrum* (1.62%), *Corynebacterium* (1.51%), and *Campylobacter* (1.31%) (Fig. 1B).

### Microbiome diversity of breast cancer tumor tissue

**Fig 2:**
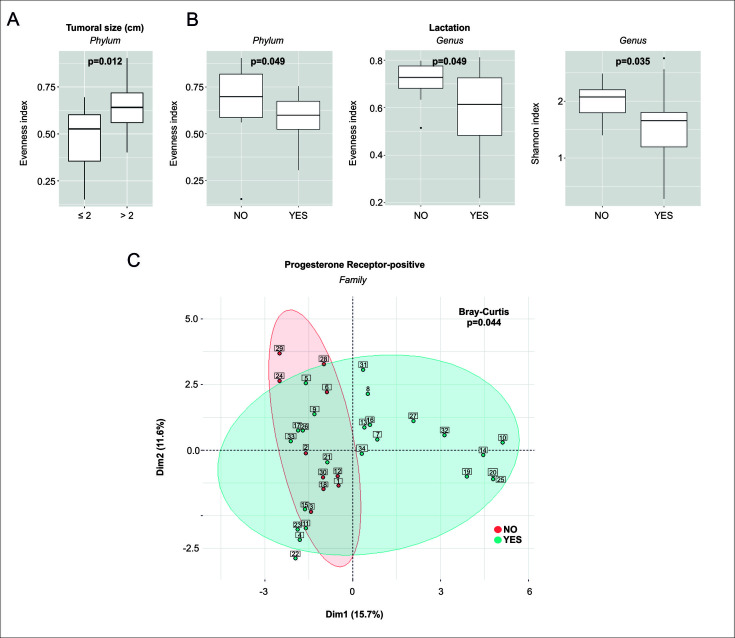
Alpha-diversity and beta-diversity comparisons show markedly different representations of the breast cancer microbiome by features evaluated. (**A**) Alpha-diversity analyses showed that tumors >2 cm had higher Evenness index values at the bacterial phylum level by a Wilcoxon test (*P* = 0.012). (**B**) Alpha-diversity analyses showed that tumors from patients with no history of lactation had greater Evenness index values at the phylum level by a Wilcoxon test (*P* = 0.049) and greater Evenness index and Shannon index values at the genus level by a Wilcoxon test (*P* = 0.049 and *P* = 0.035, respectively). (**C**) Beta-diversity analysis: principal coordinate analysis of Bray-Curtis dissimilarity indices showed significant differences between progesterone receptor-positive and progesterone receptor-negative tumor oncobiome, *P*-values were calculated using ADONIS (*P* = 0.044).

To quantify overall differences in breast microbial (composition) diversity within each sample, the alpha diversity measure was used in association with BC patient features. At the phylum level, it was found that both patients with tumors size >2 cm (Fig. 2A) and women without lactation history (Fig. 2B) showed greater uniformity in the distribution with the opposite group (Evenness index *P* = 0.012 and *P* = 0.049, respectively). However, no significant differences were found in bacterial richness and diversity, measured using the Chao 1 and Shannon indexes. At the genus level, it was found that patients without a lactation history exhibited greater bacterial uniformity and diversity (Evenness index *P* = 0.049 and Shannon index *P* = 0.035, respectively). We calculated the sample-to-sample variation by the Bray-Curtis index and visualized it using the principal coordinates samples plot of Beta diversity. We found no significant differences between the bacterial composition present in the samples in association with BC patient clinicopathological features, lifestyle-related, and survival when using phyla and genera levels. However, beta diversity analysis reveals a significant difference in PR-positive tumors at a family level, as indicated by the Bray-Curtis index (Bray-Curtis index *P* = 0.044) (Fig. 2C).

### Specific bacterial phyla and genera correlated with some breast cancer and progression disease features

**Fig 3:**
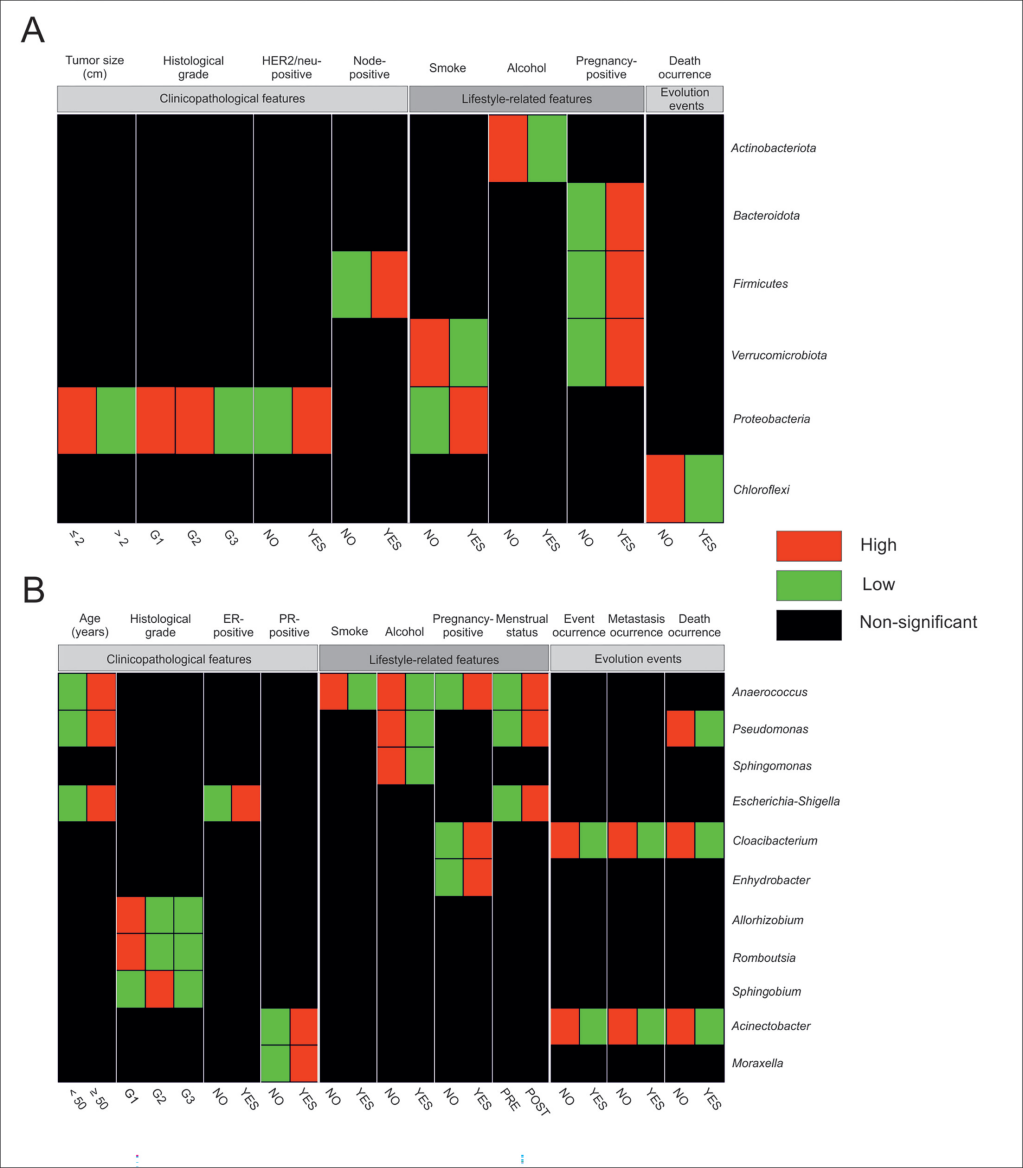
Heatmap profiles showed significantly different microbial profiles at the phylum and genus level associated with some breast cancer characteristics. The colors for each point in the heat maps indicate either higher levels (red) or lower levels (green). (**A**) Mean relative abundances of the bacterial phyla *Actinobacteria*, *Bacteroidota*, *Firmicutes*, *Verrucomicrobiota*, *Proteobacteria,* and *Chloroflexi* showed significant differences with tumor size, histological grade, and Her2/neu (human epidermal growth factor receptor 2), node, smoke, alcohol, pregnancy, and death. (**B**) Mean relative abundances of bacterial genera *Anaerococcus*, *Pseudomonas*, *Sphingomonas*, *Escherichia-Shigella*, *Cloacibacterium*, *Enhydrobacter*, *Allorhizobium*, *Romboutsia*, *Sphingobium*, *Acinetobacter,* and *Moraxella* showed significant differences with age, histological grade, ER, PR, smoke, alcohol, pregnancy, menstrual, event, metastasis, and death.

The relative abundance of bacterial phyla was investigated in relation to BC characteristics. Some of the clinicopathological features showed significant differences in the relative abundance of phyla. Patients with tumor size ≤2 cm, HER2/neu positive status, and histological Grade G2 showed a high relative abundance of *Proteobacteria* (*P* = 0.018, *P* = 0.035, and *P* = 0.040, respectively). Moreover, tumors from sentinel lymph node-positive patients were significantly associated with *Firmicutes* (*P* = 0.048) (Fig. 3A).

On the other hand, tumors from patients with a history of pregnancy exhibited a high abundance of *Bacteroidota* (*P* = 0.014), *Firmicutes* (*P* = 0.016), and *Verrucomicrobiota* (*P* = 0.036), while patients without an alcohol consumption history showed a high relative abundance of *Actinobacteriota* (*P* = 0.044). Moreover, patients with a history of smoke consumption were associated with *Proteobacteria* (*P* = 0.003), whereas those without a history of smoke consumption were significantly associated with *Verrucomicrobiota* (*P* = 0.036) (Fig. 3A).

Finally, tumors from surviving patients exhibited a high relative abundance of the *Chloroflexi* phylum (*P* = 0.035) (Fig. 3A).

The relative abundance of bacterial genera was also investigated in relation to BC characteristics. Some of the clinicopathological features showed significant differences in the relative abundance of genera.

In relation to the clinicopathological characteristics of BC, tumors from patients ≥50 years old showed significantly elevated abundance of *Anaerococcus (P* = 0.031), *Escherichia-Shigella* (*P* = 0.044), and *Pseudomonas* (*P* = 0.032) compared to the opposite group (Fig. 3B). Histological grade 1 tumors exhibited high abundance of *Allorhizobium-Neorhizobium-Pararhizobium-Rhizobium* (*P* = 0.041) and *Romboutsia* (*P* = 0.033), whereas histological grade 2 tumors were characterized by the predominance of *Sphingobium* (*P* = 0.044) (Fig. 3B). PR-positive tumors expressed high abundance of *Acinetobacter* (*P* = 0.038) and *Moraxella* (*P* = 0.044), and ER-positive tumors showed high abundance of *Escherichia-Shigella* (*P* = 0.044) (Fig. 3B). Regarding the lifestyle-related features, samples from patients with a history of pregnancy were characterized by a high relative abundance of *Anaerococcus* (*P* = 0.031), *Cloacibacterium* (*P* = 0.020), and *Enhydrobacter* (*P* = 0.046) (Fig. 3B). In post-menopausal patients, *Anaerococcus* (*P* = 0.031), *Escherichia-Shigella* (*P* = 0.044), and *Pseudomonas* (*P* = 0.007) predominated (Fig. 3B). In patients without history of alcohol consumption, the genera *Anaerococcus* (*P* = 0.031), *Pseudomonas* (*P* = 0.017), and *Sphingomonas* (*P* = 0.020) significantly predominated over the opposite group (Fig. 3B). Also patients without history of smoke consumption had a significant abundance of *Anaerococcus* (*P* = 0.037) (Fig. 3B). Finally, regarding the parameters of disease progression, it was found that the genus *Acinetobacter* (*P* = 0.036) and *Cloacibacterium* (*P* = 0.044) significantly predominated in patients without any events (Fig. 3B). Moreover, in patients without metastasis, the genus *Acinetobacter* (*P* = 0.048) and *Cloacibacterium* (*P* = 0.046) also predominated, as well as in survival patients (*Acinetobacter P* = 0.036, *Cloacibacterium P* = 0.037) (Fig. 3B).

### Specific KEGG metabolic pathways correlated with some breast cancer features

**Fig 4:**
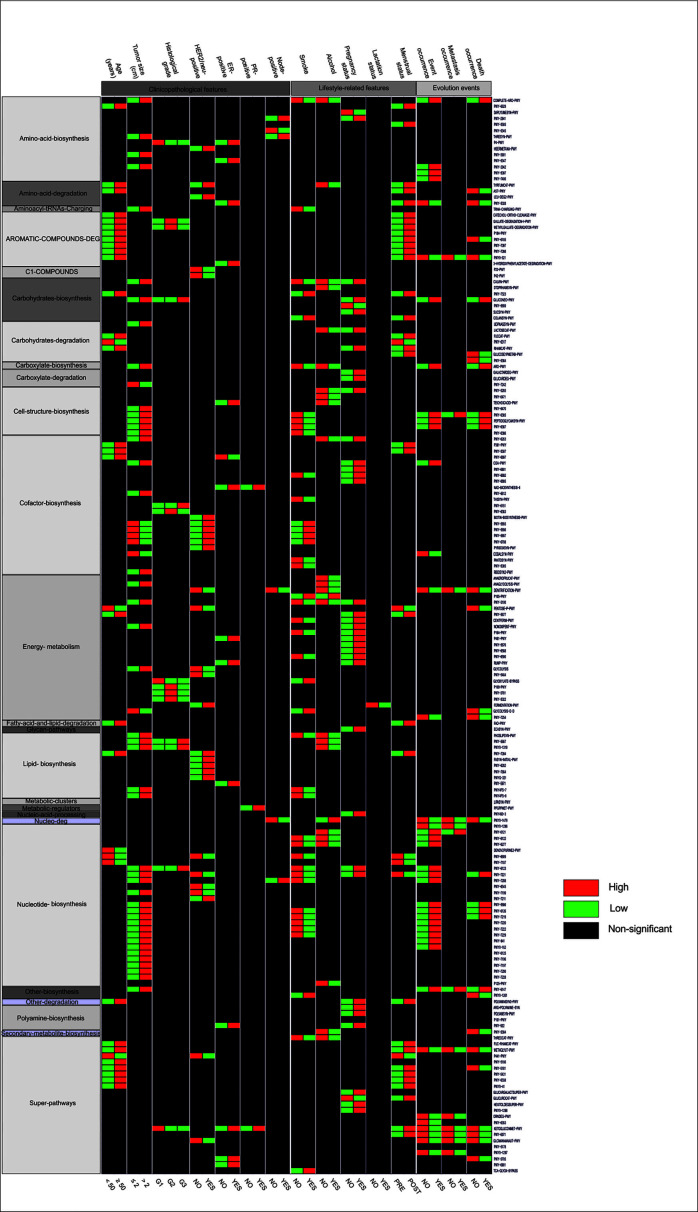
Heatmap profiles showed specific KEGG metabolic pathways correlate with breast cancer characteristics at the phylum and genus level. A total of 178 hypothetical metabolic pathways, grouped into 25 classes, were significantly associated with some clinicopathological features of breast cancer, as well as with lifestyle and patient outcomes. In particular, many pathways related to the biosynthesis of amino acids, nucleotides, and cellular structures were more highly enriched in tumors larger than 2 cm and in patients who experienced events such as local recurrence, metastasis, and/or death.

A total of 399 hypothetical (pathways) were found, which were subclassified into 33 classes according to the Metacyc database. We found that some classes are enriched in metabolic pathways (≥18 paths), such as cofactor-biosynthesis (63 paths), super-pathways (52 paths), energy-metabolism (46 paths), amino-acid-biosynthesis (34 paths), nucleotide-biosynthesis (30 paths), lipid-biosynthesis (19 paths), and carbohydrates-biosynthesis (18 paths). It was found that 236 paths showed significant differences for certain BC features. From the total predicted pathways significantly associated with BC characteristics, 178 pathways grouped into 25 classes were selected that could be linked to many clinicopathological features, lifestyle-related, and BC progression (Fig. 4). Overall, we found that pathways associated with amino-acid, nucleotide, and cell-structure biosynthesis are enriched in tumors >2 cm and with the occurrence of any event and death (Fig. 4).

## DISCUSSION

The discovery of a distinct microbiome in breast cancer (BC) tissue, first reported by Xuan et al. and Urbaniak et al. (13, 14), revealed unique and diverse bacterial profiles in tumor tissue that differ from those in healthy breast tissue, suggesting a potential role of certain bacterial species in shaping the tumor microenvironment and driving oncogenesis. Building on this, Kovács et al. (11) reinforced the concept of oncobiosis, describing a state of microbial dysbiosis specifically associated with cancer. In the context of BC, this oncobiotic state reflects an imbalance in the microbial community within tumor tissue that may facilitate tumor progression and metastasis (11). The presence of a dysbiotic microbiome in BC tissues has since been linked to cancer subtypes, immune signatures, prognostic factors, and clinical potential (15–19). Even with all these findings, the role of resident bacteria in breast carcinogenesis remains unclear. Further studies are essential to clarify the contribution of the tumor-associated microbiome to the development and progression of breast cancer. Despite the oncobiome being characterized by low biomass (10), we could study the microbiome of frozen primary tumors obtained sterilely from women with BC. We identified a distinct oncobiome in each of the samples. In relation to previous reports (13, 14, 20, 21), in our samples, the most abundant phylum included *Proteobacteria*, followed by *Firmicutes*, *Bacteroidota*, and *Actinobacteria*, which together represented more than 90% of the total phyla. Moreover, we found that *Chloroflexi*, *Fusobacteriota*, and *Campylobacterota* were the phyla that followed in the ranking (22, 23). Although normal and tumor breast tissue shares some phyla, when comparing our results with healthy breast tissue (15), we found a differential abundance of *Fusobacteriota*. Interestingly, members of the *Fusobacteriota* phylum, such as *F. nucleatum*, have been reported to have pro-carcinogenic properties (24).

At the genus level, the microbiome within the tumor has been reported to differ from the microbiome of healthy breast tissue (13–16, 18, 25). Bacteria of the genera *Lactococcus*, *Streptococcus*, *Prevotella*, *Corynebacterium*, *Micrococcus*, *Anaerococcus,* and *Propionibacterium* (14, 16, 18), have been shown to predominate in healthy breast tissue, while in women with BC, an increase in the content of *Staphylococcus*, *Acinetobacter*, *Pseudomonas*, *Brevundimonas*, *Sphingomonas*, *Aerococcus,* and *Fusobacterium* has been reported (15–17). In agreement with these data, our breast tumor tissue shows a relatively higher abundance of genera described above. Particularly, *Pseudomonas*, *Acinetobacter*, and *Staphylococcus* account for more than 23% of the total. Interestingly, *Staphylococcus epidermidis*, belonging to the genus *Staphylococcus* was isolated from breast cancer patients, had the ability to induce double-stranded DNA breaks in HeLa cells *in vitro*, and the accumulation of such damage may lead to genomic instability and malignant transformation of cells (14). Moreover, in concordance with other authors, we found the presence of the genus *Fusobacterium* in breast tumor tissue. Interestingly, this genus has been reported to have significant carcinogenic potential and to be associated with colon tumors (24). Even more, the treatment of mice bearing a colon cancer xenograft with the antibiotic metronidazole reduced *Fusobacterium* load, cancer cell proliferation, and overall tumor growth. These observations argue for further investigation of antimicrobial interventions as a potential treatment for patients with *Fusobacterium*-associated breast cancer (24).

We aimed to evaluate whether this state of dysbiosis varies in relation to each characteristic of BC. At the phylum level, we found a loss of Evenness index in tumors ≤2 cm, suggesting that the bacterial diversity of the oncobiome changes as tumor size increases. Similarly, we found a loss of Evenness (phylum and genus levels) and Shannon (genus level) indexes bacterial diversity in tumors of patients with a history of lactation. The bacterial composition of the microbiome, and therefore the oncobiome, may be affected by this process. Additionally, we observed significant dissimilarity in beta diversity at the family level between PR-negative and PR-positive tumors, indicating differences in microbial composition between both groups. These findings suggest that the presence or absence of PR expression leads to distinct tumor microenvironments, which may favor the presence of specific bacteria. Taking this into account and considering that oncobiome composition indicates dysbiosis, and it is known that dysbiosis can influence the oncogenic process and, therefore, disease progression, as well as affect the efficacy of antineoplastic therapies (11), we suggest that dysbiosis may vary according to tumor size, patients’ breastfeeding history, and hormonal receptor status.

We performed an association analysis between the abundance of bacterial taxa and BC features. This study shows differences in bacterial community and composition when comparing the oncobiome with the clinicopathological data and lifestyle-related features, as well as disease progression events. We observed that *Proteobacteria* was positively associated with tumors ≤2 cm and negatively associated with G3 histological grade. Interestingly, a study conducted on adrenocortical carcinoma tumors found that low abundance of *Proteobacteria* was negatively associated with tumor size (26). These results suggest that the abundance of *Proteobacteria* is strongly associated with small size and low histological grade, indicating a protective factor against tumor progression. Moreover, we found that *Firmicutes* phylum was significantly associated with node-positive status. Our finding suggests a relationship between the abundance of *Firmicutes* and the lymphatic spread of the disease. However, it is necessary to find more robust evidence to be more conclusive about these findings. Furthermore, we found that the *Chloroflexi* phylum was positively associated with survival in patients, suggesting new evidence of a protective role of this phylum in BC evolution. Future studies should be conducted to further understand these results.

At the genus level, we also observed a positive association with several BC features. Interestingly, we found that *Escherichia-Shigella* was significantly expressed in tumors of ER-positive. These bacteria have also been shown to have β-glucuronidase activity, which promotes the up-regulation of estrogen (27). In addition, there is a hypothesis that estrogen binding to β-glucuronidase may be related to microbiota dysregulation in BC patients (27). According to Chan et al. (28), higher levels of β-glucuronidase were observed in the nipple aspirate fluid of BC patients compared to healthy women (28). This indicates that BC oncobiome has β-glucuronidase activity, which may contribute to the development of the disease. We suggest that it would be important to assess the β-glucuronidase activity in ER-positive tumors, as stimulation of the receptor may contribute to poor disease progression. Results showed that *Cloacibacterium* was positively associated with survival in patients. Tzeng et al. (16) found that *Cloacibacterium* was present as nodes in both microbiome—immune gene and microbiome—cytokine networks, though they formed fewer connections with immune features (16). This genus may influence the role of the oncobiome in tumor development.

From a functional perspective, oncobiome metabolite signaling is recognized as a crucial regulator of BC progression. Bacteria secrete metabolites and toxins that exert multifaceted effects on the BC microenvironment, impacting cell growth, proliferation, metabolism, DNA stability, epithelial-mesenchymal transition, metastasis, and modulation of antitumor immune responses (11).

In our predictive analysis of metabolic pathways, we identified tumors enriched in genes associated with key functional categories characteristic of the oncobiome. Notably, many of these pathways exhibited associations with various BC features, encompassing clinicopathological parameters, lifestyle-related factors, and over-survival. It has been reported that pathways governing energy metabolism, amino acid, and nucleotide biosynthesis have been implicated in driving cell proliferation (29). In particular, we found pathways associated with nucleotide biosynthesis were notably enriched in tumors >2 cm and positively correlated with tumor bad progression events. Accompanying this action, we found increased amino-acid biosynthesis in tumors >2 cm and patients with poor outcomes. Furthermore, we found increased amino acid degradation in the tumors of surviving patients. This finding is an indication that tumor cells are taking advantage of this effect of the microbiome during tumor progression. Adding to our results, we also found that pathways involved in cell-structure biosynthesis and lipid metabolism were enriched in larger tumors and associated with tumor progression events. Increased phospholipid and lipopolysaccharide biosynthesis, are known drivers of cell proliferation, migration, and metastasis, underscoring their potential roles in BC (11, 28).

Finally, these findings highlight the complexity and relevance of the oncobiome in breast cancer. However, limitations inherent to this type of research, such as the low microbial biomass and potential biases in sampling and analytical techniques, must be addressed in future studies. Longitudinal and experimental research will also be essential to validate these associations and explore clinical applications, such as diagnostic biomarkers and microbiome-targeted therapies.

Together, our findings provide deeper insights into the role of the microbiome in BC, opening new avenues for future research and therapeutic applications.

### Conclusions

The inclusion of a diverse Argentine BC patient cohort enriches the existing knowledge base, reflecting the global significance of this research.

In perspective, the results emphasize the necessity of further research in this field to enhance our understanding of the microbiome’s role in BC and to potentially identify novel therapeutic targets or prognostic indicators. Understanding the complex interplay among the microbiome, tumor microenvironment, and BC pathogenesis holds promise for more personalized and efficient treatment approaches in the future.

## Data Availability

All sequencing data have been uploaded to the National Center of Biotechnology (NCBI) and are available under the accession number PRJNA1197483.
